# Impact of endoscopic ultrasound‐guided transmural drainage for postoperative pancreatic fistula after pancreatic surgery

**DOI:** 10.1002/deo2.270

**Published:** 2023-07-02

**Authors:** Ryoichi Miyamoto, Amane Takahashi, Toshiro Ogura, Kei Kitamura, Hiroyuki Ishida, Shinichi Matsudaira, Yuko Suzuki, Satoshi Shimizu, Yoshiyuki Kawashima

**Affiliations:** ^1^ Department of Gastroenterological Surgery Saitama Cancer Center Saitama Japan; ^2^ Department of Gastroenterological Surgery Tokyo Medical University Ibaraki Medical Center Ibaraki Japan; ^3^ Department of Gastroenterology Saitama Cancer Center Saitama Japan

**Keywords:** EUS‐guided drainage, pancreatic fistula, pancreatic fluid collections, pancreatic surgery, POPF

## Abstract

**Objectives:**

Postoperative pancreatic fistula (POPF) is a major cause of morbidity after pancreatic surgery. Recently, endoscopic ultrasound‐guided transmural drainage (EUS‐TD) has been widely used to manage pancreatic pseudocysts after acute pancreatitis. Several studies have reported the effectiveness of EUS‐TD for POPF, although there is insufficient evidence regarding the performance of EUS‐TD for POPF. We herein report on the safety, efficacy, and appropriate timing of EUS‐TD for POPF compared with conventional percutaneous intervention.

**Methods:**

Eight patients who underwent EUS‐TD of POPF and 36 patients who underwent percutaneous intervention were retrospectively enrolled. Clinical outcomes, including technical success, clinical success, and complications, were analyzed among the two groups.

**Results:**

In terms of clinical outcomes between the EUS‐TD and percutaneous intervention groups, significant differences were observed in the number of interventions (1 vs. 4, *p* = 0.011), period of clinical success (6 days vs. 11 days, *p* = 0.001), incidence of complications (0 vs. 3, *p* = 0.021), postoperative hospital stays (27 days vs. 34 days, *p* = 0.027), and recurrence of POPF (0 vs. 5, *p* = 0.001).

**Conclusions:**

EUS‐TD for POPF appears to be safe and technically feasible. This approach should be considered a therapeutic option in patients with POPF after pancreatic surgery.

## INTRODUCTION

Postoperative pancreatic fistula (POPF) is a major cause of morbidity after pancreatic surgery, with a frequency ranging from 2% to 20%. POPF may be asymptomatic, but some can lead to a hemorrhage of the splenic or gastroduodenal artery stump, severe pain, gastric outlet obstruction, fistulas, intraabdominal infection, and sepsis.^1^, ^2^

Traditionally, pancreatic fluid collections (PFCs) have been managed by percutaneous or operative drainage. Recently, endoscopic ultrasound‐guided transmural drainage (EUS‐TD) has been widely used to manage pancreatic pseudocysts or walled‐off necrosis lesions after the onset of acute pancreatitis. EUS‐TD is usually performed 4 weeks after the onset of acute pancreatitis to allow for encapsulation of these lesions so that the margins of PFCs can be clearly defined on imaging.^3^, ^4^ EUS‐TD for POPF was initially recognized as an alternative treatment modality for cases in which percutaneous drainage was ineffective or technically difficult to perform. Varadarajulu initially reported EUS‐TD for the management of PFCs after distal pancreatectomy in 10 patients with a high technical and clinical success rate with fewer associated complications in 2009.^5^ Currently, several studies have recommended internal intervention as the first‐line treatment for managing PFCs, including POPF, considering the many advantages over percutaneous intervention.^6^, ^7^, ^8^, ^9^

However, there is insufficient evidence regarding the safety and efficacy of performing EUS‐TD for POPF compared to percutaneous intervention. Furthermore, the optimal timing and device for performing EUS‐TD for POPF have not been fully elucidated, and only a few reports have specifically focused on the outcomes of early, postoperative drainage procedures.^10^, ^11^

We herein report on the safety, efficacy, and appropriate timing of EUS‐TD for POPF compared with percutaneous intervention.

## MATERIALS AND METHODS

### Patients

A retrospective cohort of 163 patients who underwent pancreatic surgery at Saitama Cancer Center between January 2020 and December 2022 were enrolled. The ethics committee of Saitama Cancer Center approved this study. POPF was defined in accordance with the guidelines of the International Study Group on Pancreatic Fistula.^12^ Instances of POPF were also defined as grade B or C.

First, we excluded 118 patients without POPF or with grade A POPF. Regarding the adaptation of EUS‐TD, patients were chosen for EUS‐TD based on their imaging studies and the consensus opinion of the surgeon and endoscopist. Furthermore, EUS‐TD was employed as an alternative treatment modality for cases in which percutaneous drainage was ineffective or technically difficult to perform. Among 45 patients with grade B POPF, 36 patients were indicated for percutaneous intervention, while eight patients were indicated for EUS‐TD. Only one patient who received both percutaneous intervention and EUS‐TD was excluded from the present study. Among the two groups, we analyzed the short‐term and long‐term outcomes of patients who underwent EUS‐TD compared with percutaneous intervention.

### EUS‐TD procedure

The technique of the EUS‐TD procedure has been described in detail in previous literature by Ang TL et al.^3^ A convex array echoendoscope (GF‐UCT240‐AL5; Olympus Medical Systems, Tokyo, Japan), a US system (ProSound SSD‐5000; ALOKA, Tokyo, Japan), and a fluoroscope (CUREVISTA; Hitachi Medical Corp., Tokyo, Japan) were used for all the procedures.

**FIGURE 1:**
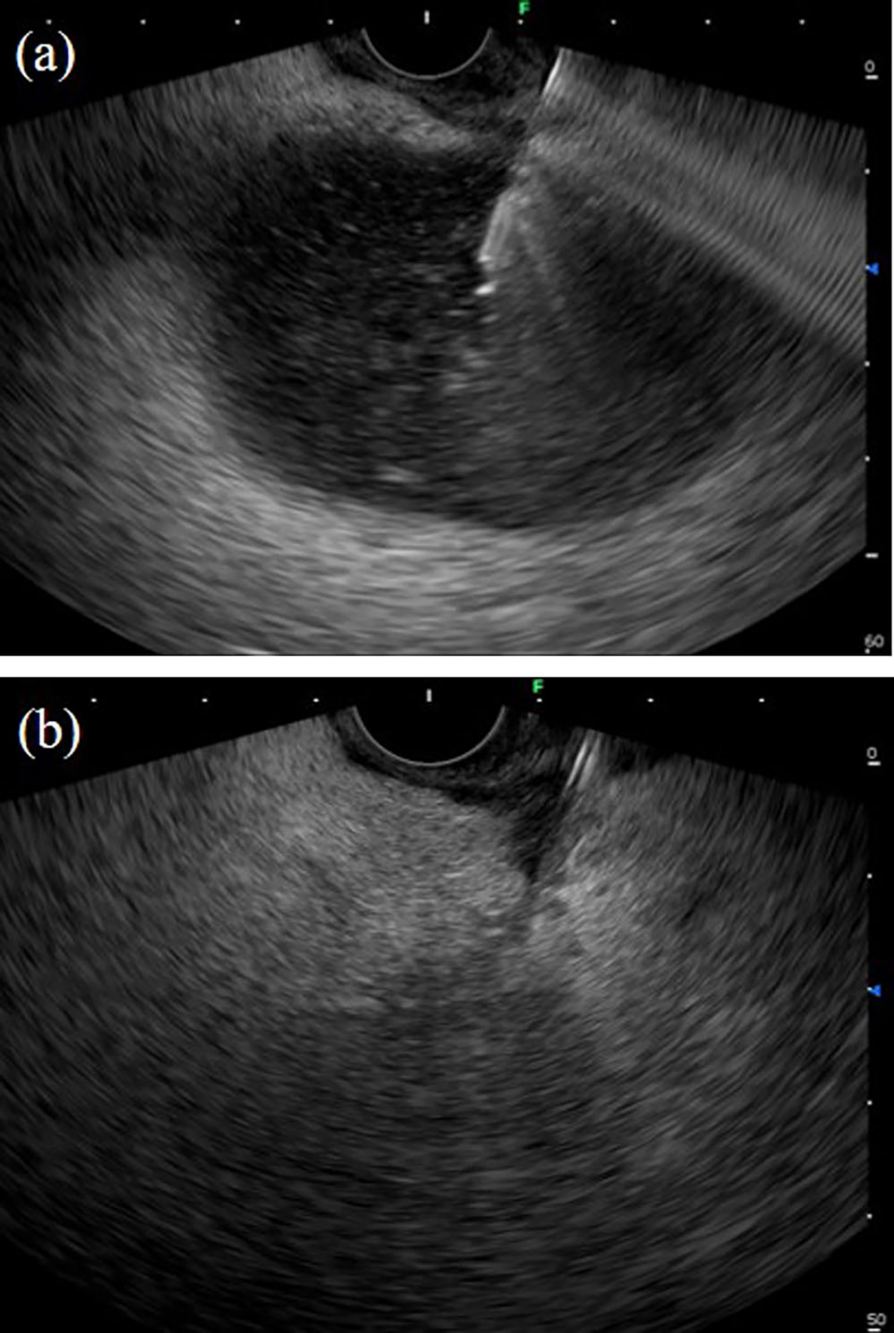
(a) The pancreatic fluid collection was identified by ultrasound. A 19‐gauge fine needle aspiration needle was used to puncture through the gastric wall into the pancreatic fluid collection. (b) After insertion of a 19‐gauge needle into the pancreatic fluid collection under endoscopic ultrasound guidance, the fluid within was aspirated.

The fluid collection was identified by ultrasound, and Doppler flow was used to interrogate the field to ensure the absence of vasculature in the needle tract. When blood vessels were located close to the fluid collection, every effort was made to find an optimal location several centimeters away from these vessels. A 19‐gauge fine needle aspiration needle (Echo‐tip Ultra, Cook Medical, Winston Salem, NC) was used to puncture through the gastric wall or enteral wall into the fluid collection (Figure 1). After insertion of a 19‐gauge needle into the PFCs under EUS guidance, the fluid within was partially aspirated for bacteriological culture analysis and evaluation of its amylase level. Whenever plastic stent placement was intended, a 0.025‐inch guidewire was inserted and looped into the cavity. Thereafter, the fistula tract was dilated using either a no‐cauterizing device (Hurricane RX or CRE, Boston Scientific, Natick, MA; Ren, Kaneka, Tokyo, Japan; ES dilator, Zeon Medical Inc., Tokyo, Japan) or a cautery (Cysto‐Gastro‐Set; Endo‐Flex GmbH, Voerde, Germany). This was followed by the insertion of a 7‐Fr size, double‐pigtailed plastic stent, and/or a 5‐6‐Fr nasocystic drainage tube along the guidewire into the lumen. Patients who were clinically improved at 4–6 weeks were reassessed by multidetector computed tomography (MDCT) to confirm the resolution of the PFCs and then were scheduled to undergo endoscopic removal of the stents.

### Percutaneous intervention

The percutaneous intervention included percutaneous drainage and drain replacement procedures. We intraoperatively used drainage tubes, which were placed superiorly at the anastomotic site between the pancreas and the jejunum or the pancreatic stump. In terms of percutaneous drainage, using real‐time ultrasound or CT guidance, an 18‐gauge needle was punctured percutaneously into the PFCs, and fluid was aspirated. A guide wire (Safe‐T J Curved, COOK, USA) of 0.035‐inch in diameter was advanced into the collection, and the tract was sometimes dilated. Then, either a 7.5‐ or 8.0‐Fr pigtailed drainage catheter was placed into the fluid collection. The collection was emptied as completely as possible, and then post‐drainage imaging was performed. Catheter exchange or removal was based on clinical improvement as well as drainage catheter output, catheter malfunction or dislodgement, and evidence of persistent fluid on repeat imaging. Catheter removal was at the discretion of the surgeon.

In terms of drain replacement procedures, the volumes of the drained fluid were measured every day, and their amylase levels were monitored on postoperative days 1, 3, 5, and 7. The drainage tubes were removed on POD 4–5 for patients who were judged as having an absence of POPF or grade A POPF. In possible POPF grade B or C patients, regardless of the low concentration of drainage amylase levels, drainage tubes were replaced with new 14–18‐Fr silicon tubes (Fuji Systems Co., Tokyo, Japan). We performed drain imaging and replacement once a week and examined the relationships between the drain tract and the space of PFCs. If the fistula connected to the digestive tract or only the drain tract was observed, the drain was removed.

### Type of PFCs classified by MDCT findings

**FIGURE 2:**
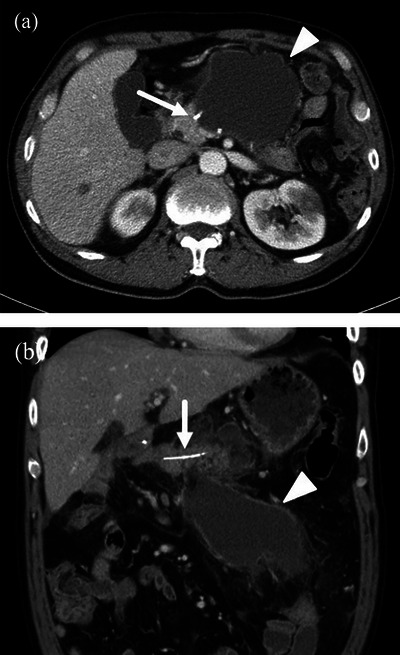
(a) Axial multidetector computed tomography view in the patient who underwent distal pancreatectom is shown. The arrow indicates the pancreatic stump by the linear stapler, and the arrowhead indicates the pancreatic fluid collection with an encapsulated wall. (b) Reconstructed coronal multidetector computed tomography view in the patient who underwent subtotal stomach‐preserving pancreatoduodenectomy is shown. The arrow indicates the internal stent in the pancreatojejunostomy, and the arrowhead indicates the pancreatic fluid collection with an encapsulated wall.

**FIGURE 3:**
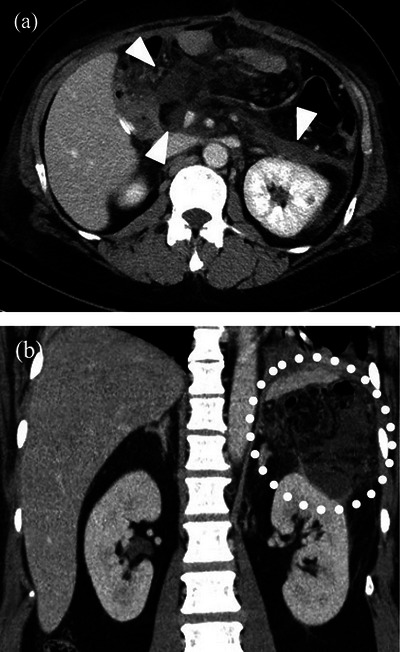
(a) Axial multidetector computed tomography view in the patient who underwent stomach‐preserving pancreatoduodenectomy is shown. Arrowheads indicate the pancreatic fluid collections without encapsulated walls. (b) Reconstructed coronal multidetector computed tomography view in the patient who underwent distal pancreatectomy is shown. The area enclosed by dotted lines indicates pancreatic fluid collections without encapsulated walls.

In our study, we originally classified the type of PFC by MDCT findings. If we suspected the presence of PFCs, we routinely performed an MDCT examination before EUS‐TD or percutaneous intervention. Based on the characteristic MDCT findings, the shape of the PFCs was classified into the following three types: PFC with cystic type was defined as a cystic collection with an encapsulated wall around the gastrointestinal tract (Figure 2). Diffused‐type PFCs were defined as spread diffusely without an encapsulated wall (Figure 3). Mixed‐type PFCs were defined as a combined image of both cystic‐type and diffused‐type PFCs.

### Definitions of clinical outcomes

Technical success for EUS‐TD was defined as the ability to successfully localize the PFC by EUS, perform a needle puncture, aspirate fluid, or deploy a stent. Technical success for percutaneous intervention was defined as the ability to successfully localize the PFCs by imaging, performing a needle puncture, aspirate fluid, and placing or replacing a percutaneous drainage catheter. Clinical success was defined as the resolution of the PFCs on radiological findings and the resolution of symptoms, including fever, leukocytosis, pain, nausea, or vomiting.

Outcomes included technical success, clinical success, number of interventions, period of clinical success, fluid amylase level, bacterial culture in drainage collection, complications excluding the presence of POPF, and postoperative hospital stays. Furthermore, long‐term outcomes, including the recurrence of POPF, were also analyzed. The postoperative complications were also graded according to the Clavien‒Dindo classification.^13^

### Statistical analyses

Correlations with the patients’ background data were analyzed using a *χ*2 test or Fisher's exact test, as appropriate. Similarly, correlations with the clinical outcomes were also analyzed using a *χ*2 test or Fisher's exact test, as appropriate. Statistical analyses were performed using a statistical analysis software package (SPSS Statistics, version 21; IBM, Armonk, NY, USA), and *p*‐values < 0.05 were considered significant.

## RESULT

### Clinical summary of the patients who underwent EUS‐TD (Table 1)

Eight patients with a median age of 61 years (range 37–84 years) underwent EUS‐TD for grade B POPF; the median follow‐up period was 24.8 months (range 8.06–33.0 months). Primary diseases consisted of three patients with pancreatic duct cancer, one with mucinous cyst adenoma, one with intraductal papillary mucinous neoplasm, one with duodenum cancer, one with pancreatic neuroendocrine tumor, and 1 with biliary duct cancer. Surgical procedures consisted of five subtotal stomach‐preserving pancreatoduodenectomies, 1 laparoscopic distal pancreatectomy, one robot‐assisted distal pancreatectomy, and one hepato‐pancreaticoduodenectomy. Six patients were treated with one EUS‐TD puncture. However, the other two patients needed revision for the transmural deployment of the stent or drainage tube. The route of drainage was transgastric in five patients, transjejunal in one patient, and both transgastric and transjejunal in two patients. The median duration of the EUS‐TD procedure was 35.5 minutes (range 17–77 min). The reasons why the procedure time of EUS‐TD took so long were as follows. Multiple punctures were required due to multiple PFCs. In order to determine the puncture route, such as postural change, there were blood vessels in the puncture route. In terms of case 7, because it was a recurrence after fine needle aspiration, it took a long time to deployment of a stent and nasocystic drainage tube. In terms of the PFC types, there were six patients with the cystic type, 1 with the diffused type, and 1 with both the cystic and diffused types. The median size of the fluid collection before drainage was 50 mm (range 31–120 mm). The median time from operation to drainage was 16 days (range 8–21 days).

**TABLE 1:** Characteristics of patients who underwent endoscopic ultrasound‐guided transmural drainage for postoperative pancreatic fistula.

Case	Age/sex	Disease	Operation	No. of EUS‐TD procedures	Drainage approach	Drainage procedure	Length of procedure (min)	Type of PFC	Size of PFC (mm)	Time from operation to EUS‐TD (days)
1	61/F	PDC	SSPPD	1	Transgastric/transjejunal	Aspiration	43	Cystic	75	16
2	84/F	PDC	SSPPD	1	Transgastric/transjejunal	Aspiration	77	Cystic	45	13
3	37/F	MCA	LDP	2	Transgastric	Aspiration, aspiration	24, 60	Diffuse	31, 40	21, 23
4	65/F	IPMN	SSPPD	1	Transgastric	Aspiration/ENCD	30	Mixed	50	8
5	50/M	DC	SSPPD	1	Transjejunal	Aspiration	41	Cystic	120	19
6	57/F	PNET	SSPPD	1	Transgastric	Aspiration	17	Cystic	50	14
7	69/M	PDC	RDP	2	Transgastric	Aspiration, stent/ENCD	18, 60	Cystic	80, 80	14, 17
8	63/M	BTC	HPD	1	Transgastric	Aspiration	30	Cystic	28	18

EUS‐TD, endoscopic ultrasound‐guided transmural drainage; POPF, postoperative pancreatic fistula; PFC, pancreatic fluid colllection; PDC, pancreatic duct cancer; MCA, mucinous cystadenoma; IPMN, intraductal papillary mucinous neoplasm; DC, duodenum cancer; PNET, pancreatic neuroendocrine tumor; BTC, Biliary truct cancer; SSPPD, subtotal stomach‐preserving pancreatoduodenectomy; LDP, laparoscopic distal pancreatectomy; RDP, robot‐assisted distal pancreatectomy; HPD, hepatopancreatoduodenectomy; ENCD, endoscopic nasocystic drainage.

### Patient characteristics between the EUS‐TD and percutaneous intervention groups (Table 2)

The patient characteristics between the EUS‐TD and percutaneous intervention groups are presented in Table 2. In terms of the type of PFC and time from operation to EUS‐TD, significant differences were observed between the two groups (*p* = 0.003, *p* = 0.021).

**TABLE 2:** Comparison in patients with a postoperative pancreatic fistula treated using endoscopic ultrasound‐guided transmural drainage (EUS‐TD) and percutaneous intervention.

Factors	EUS‐TD (*n* = 8)	Percutaneous intervention (*n* = 36)	*p*‐Value
Age (years)	61 (37–84)	62 (29–83)	0.411
Male/female	3/5	22/14	0.375
Surgical procedures			
SSPPD	5	23	0.418
DP	0	8	
LDP	1	3	
Others	2	2	
Location of fluid collection			
Anastomosis of PJ or PG	4	14	0.880
Pancreatic stump	1	8	
Others	3	14	
Size of fluid collection (mm)	50 (40–120)	36 (5–117)	0.083
Type of PFCs			
Cystic	6	1	0.003*
Diffused	1	27	
Mixed	1	5	
Others	0	3	
Time from operation to intervention (days)	16 (8–21)	7 (4–14)	0.021*
No. of interventions	1 (1–2)	4 (1–16)	0.011*
Technical success (*n*, %)	10/10 (100)	111/113 (98.2)	0.676
Clinical success (*n*, %)	8/8 (100)	36/36 (100)	1.000
Period of clinical success (days)	6 (1–14)	11 (1–62)	0.001*
Fluid amylase level (mg/dL)	17,363 (1323–39,971)	23,642 (629–145,940)	0.233
Bacterial culture in drainage collection			
Positive	7	32	0.588
Negative	0	4	
Complications (C‐D grade > III)	0	3	0.021*
Postoperative hospital stay (days)	27 (15–55)	34 (18–82)	0.027*
Recurrence of POPF	0	5	0.001*
Follow‐up period (months)	24.8 (8.06–33.0)	17.4 (1.53–34.9)	0.177

POPF, postoperative pancreatic fistula; EUS‐TD, endoscopic ultrasound‐guided transmural drainage; BMI, body mass index; SSPPD, subtotal stomach‐preserving pancreatoduodenectomy; DP, distal pancreatectomy; LDP, laparoscopic distal pancreatectomy; PJ, pancreatojejunostomy; PG, pancreatogastrostomy; PFC, pancreatic fluid collections; C‐D, Clavien‐Dindo Classification.

### The clinical outcomes between the EUS‐TD and percutaneous intervention groups (Table 2)

In the EUS‐TD group, the technical success rate was 100% in 10 trials, including six patients who were successfully treated with one puncture and two patients who needed revision for the transmural deployment of the stent or drainage tube. Similarly, the clinical success rate was 100% in all 8 patients.

In the percutaneous intervention groups, the technical success rate was 98.2% in 113 trials. One patient treated with percutaneous intervention required a median of four interventions (range 1–16 times). The clinical success rate was 100% in all 36 patients. Adverse events (Clavien‒Dindo > III) were observed in three patients. Two patients developed bleeding from the splenic artery stump after distal pancreatectomy, and one patient developed bleeding from the gastroduodenal artery stump after stomach‐preserving pancreatoduodenectomies.

In terms of the short‐term outcomes between the EUS‐TD and percutaneous intervention groups, the number of interventions (1 vs. 4, *p* = 0.011), period of clinical success (6 days vs. 11 days, *p* = 0.001), presence of complications (Clavien‒Dindo > III; 0 vs. 3, *p* = 0.021) and length of postoperative hospital stay (27 days vs. 34 days, *p* = 0.027) were significantly different.

With respect to long‐term outcomes, the recurrence of POPF (0 vs. 5, *p* = 0.001) was significantly different between the two groups. For all five patients with recurrence of POPF, a percutaneous drainage tube was reinserted.

## DISCUSSION

In the present study, a shorter period of clinical success, absence of severe adverse events, shorter length of postoperative hospital stay, and absence of POPF recurrence were observed in the EUS‐TD group than in the percutaneous intervention group, so this approach should be considered as a therapeutic option in patients with POPF after pancreatic surgery.

The reason why EUS‐TD for POPF correlates with a favorable clinical outcome remains unanswered. One reason was that EUS allowed real‐time evaluation of the PFCs and ensured the absence of vasculature in the needle tract by ultrasound and Doppler flow. Previous studies reported that the EUS‐TD procedure allows access to relatively small and nonbulging pancreatic fistulas and provides visualization of the blood vessels to reduce the risk of bleeding.^13^, ^14^ The second reason was that EUS‐TD involved a puncture through the gastrointestinal tract into the abscess cavity, allowing for more efficient direct internal drainage of the abscess into the gastrointestinal tract. The EUS‐TD procedure also permits internal drainage of pancreatic fluid into the gut, and direct fistula formation between the POPF space and the gut can facilitate POPF closure in one step.^6^, ^14^, ^15^

In contrast, percutaneous intervention sometimes requires two or more procedures in a multistep conversion from external to internal drainage and necessitates a longer hospital stay than EUS‐TD. Furthermore, external drains are associated with a decreased quality of life requiring frequent maintenance, with a small risk of local infection, external fistula formation, and fluid and electrolyte losses.^16^, ^17^ In the present study, all three patients with serious complications had hemorrhage of the splenic or gastroduodenal artery stump. These phenomena were assumed to result from inadequate drainage of the abscess by the percutaneous drain.

In terms of radiological findings about PFCs, we assumed that the cystic‐type PFCs were useful for EUS‐TD. The reasons were that the cystic type could be expected to be more efficient in approaching the abscess through the gastrointestinal tract and more effective at internal drainage after a puncture. Moreover, postoperative adhesion between the PFCs and the gastrointestinal tract could aid in preventing intraperitoneal spillage of PFCs by drainage procedures. In fact, there were six patients (75%) with cystic‐type PFCs in the EUS‐TD group and one patient (2.7%) in the percutaneous intervention group.

In the present study, we originally reviewed retrospective studies about the efficacy of EUS‐TD for POPF (Table 3).^5^, ^6^, ^7^, ^8^, ^9^, ^10^, ^11^, ^18^, ^19^, ^20^ Among our 11 reviewed studies, including the present study, technical success rates ranged from 90% to 100%, and clinical success rates ranged from 79% to 100%. Furthermore, there was no rate of severe associated complications in six studies, and the rate of severe complications, including perforation, bleeding, and pancreatitis, ranged from 6% to 14% in the other 5 studies. These data suggest that the EUS‐TD of POPF is safe and technically feasible. Kwon et al. and Tilara et al. reported that patients with radiographic evidence of tissue necrosis or solid debris should undergo endoscopic necrosectomy either at the time of initial cyst‐gastrostomy or be considered for early repeat endoscopy with necrosectomy if drainage alone fails to achieve clinical success. It is also preferable to place multiple stents because fluid and debris appear to drain not only through the stents but also through the space between the stents in the cyst‐gastrostomy tract.^7^, ^8^ In fact, cases 3 and 7, whose PFCs were suspected to contain tissue necrosis or solid debris, required early repeat endoscopy with aspiration or deployment of a stent and nasocystic drainage tube.

**TABLE 3:** Retrospective studies about patients who underwent endoscopic ultrasound‐guided transmural drainage (EUS‐TD) for postoperative pancreatic fistula.

Author	Year	*N*	Surgical procedures	Technically success (%)	Clinically success (%)	Complications (%)	Days from operation to EUS‐TD
Varadarajulu et al.^5^	2009	10	DP	100	90	10	36 (11–350)
Varadarajulu et al.^18^	2011	20	DP	100	100	0	55 (10–118)
Gupta et al.^19^	2012	23	ND	100	79	14	ND
Onodera et al.^6^	2012	6	PD, DP	100	100	0	135 (30–720)
Kwon et al.^7^	2013	9	DP, enucleation	100	100	0	ND
Tilara et al.^8^	2014	29	PD, DP	100	93	6	63 (5–547)
Futagawa et al.^9^	2017	12	ND	92	92	0	15 (10–44)
Jürgensen C et al.^11^	2019	49	PPPD, PD	90	96	0	15 (5–144)
Wang et al.^20^	2021	15	PD, DP	100	93	7	10 (5–32)
Fujimori et al.^10^	2021	30	DP, SPDP, enucleation, others	100	97	7	17 (3–232)
The present study	2022	8	SSPPD, LDP, RDP, HPD	100	100	0	16 (8–21)

EUS‐TD, endoscopic ultrasound‐guided transmural drainage; POPF, postoperative pancreatic fistula; *N*, number; DP, distal pancreatectomy; ND, not described; PD, pancreatoduodenectomy; PPPD, pylorus‐preserving pancreaticoduodenectomy; SPDP, spleen‐preserving distal pancreatectomy; SSPPD, subtotal stomach‐preserving pancreatoduodenectomy; LDP, laparoscopic distal pancreatectomy; RDP, robot‐assisted distal pancreatectomy; HPD, hepatopancreatoduodenectomy.

The appropriate time for choosing EUS‐TD remains controversial. Most studies excluded patients with PFCs within 4 weeks after surgery because of the presumed lack of a mature wall.^21^, ^22^, ^23^ Similarly, endoscopists tend to hesitate to perform EUS‐TD during the early postoperative period, considering the immaturity of adhesions between POPF and adjoining gastric or duodenal walls and the immature encapsulated wall of PFCs.^10^ If PFCs were managed by percutaneous drainage or intervention via the drain route, the early time for drainage should be recommended. In fact, the median time for percutaneous intervention in 36 patients was 7 days after surgery. Among our 11 reviewed studies, the median time from the operative procedure to EUS‐TD was 17 days. Recent studies have focused on evaluating the success and safety of drainage completed within 4 weeks after surgery.^8^, ^10^ In fact, the median time for EUS‐guided drainage in eight patients was 16 days after surgery, and no related adverse events occurred. Unlike acute pancreatitis, we assumed that postoperative adhesions could promote the encapsulation of PFCs around the gastrointestinal tract earlier. This phenomenon could aid in preventing intraperitoneal spillage of PFCs by drainage procedures. Therefore, we thought that it was too late to wait for the capsule wall to mature because of the acute severe presentation and subsequent life‐threatening complications, including hemorrhage. Early drainage quickly relieves clinical symptoms, shortens the hospital stay, and prevents more dangerous complications.

Several limitations in EUS‐TD for POPF must be addressed. First, the present study was a retrospective single‐center study with a relatively small patient cohort, and the cooperative work with an experienced endoscopist and pancreatic surgeon was essential for carrying out EUS‐TD for POPF safely. Therefore, these results require confirmation by additional multicenter large‐scale studies and prospective randomized controlled studies.

In conclusion, EUS‐TD should be considered as a therapeutic option in patients with POPF after pancreatic surgery. In addition, the present study assumed that early drainage (<4 weeks) of postoperative PFCs was feasible and safe.

## CONFLICT OF INTEREST STATEMENT

None.
